# Temporal imaging of drug dynamics in live cells using stimulated Raman scattering microscopy and a perfusion cell culture system†

**DOI:** 10.1039/d2cb00160h

**Published:** 2022-08-09

**Authors:** William J. Tipping, Andrew S. Merchant, Rebecca Fearon, Nicholas C. O. Tomkinson, Karen Faulds, Duncan Graham

**Affiliations:** a Centre for Molecular Nanometrology, WestCHEM, Department of Pure and Applied Chemistry, Technology and Innovation Centre, University of Strathclyde Glasgow G1 1RD UK karen.faulds@strath.ac.uk duncan.graham@strath.ac.uk; b Department of Pure and Applied Chemistry, University of Strathclyde Glasgow G1 1XL UK nicholas.tomkinson@strath.ac.uk

## Abstract

Stimulated Raman scattering (SRS) microscopy is a powerful technique for visualising the cellular uptake and distribution of drugs and small molecules in live cells under biocompatible imaging conditions. The use of bio-orthogonal groups within the drug molecule, including alkynes and nitriles, has enabled the direct detection of a plethora of bioactive molecules in a minimally perturbative fashion. Limited progress has been made towards real-time detection of drug uptake and distribution into live cells under physiological conditions, despite the accordant potential it presents for preclinical drug development. SRS microscopy has been applied to the study of cellular dynamics of the drug 7RH, which is a potent inhibitor of dicoidin domain receptor 1 (DDR1) and prevents cellular adhesion, proliferation and migration *in vitro*. The uptake of 7RH into a variety of mammalian cell models was shown to be independent of DDR1 expression. Using a perfusion chamber, the recurrent treatment of live cancer cells was achieved, enabling 7RH uptake to be visualised in real-time using SRS microscopy, after which the viability of the same cellular population was assessed using commercially available fluorescent markers in a multimodal imaging experiment. The effect of 7RH treatment in combination with the chemotherapeutic, cisplatin was investigated using sequential perfusion and time-lapse imaging in the same live cell population, to demonstrate the application of the approach. SRS microscopy also identified potent inhibition of cellular adhesion and migration in breast cancer cell models with increasing 7RH treatment concentrations, thus representing a novel read-out methodology for phenotypic assays of this kind. The direct assessment of drug–cell interactions under physiological conditions offers significant potential for the preclinical drug development process.

## Abstract 2

Multimodal imaging of drug uptake and cell viability analysis in the same live cell population is enabled using a perfusion cell culture system.

## Graphical abstract

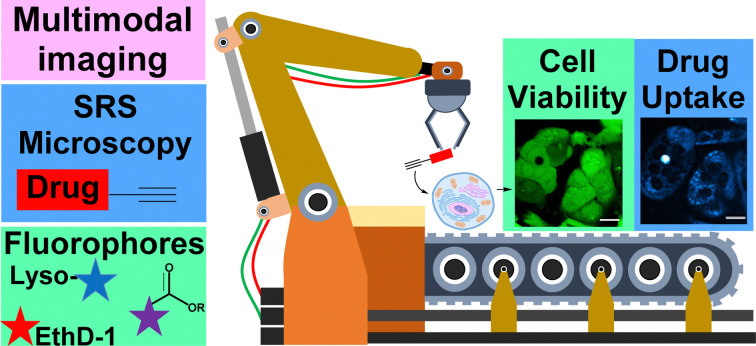

## Introduction

**Fig. 1:**
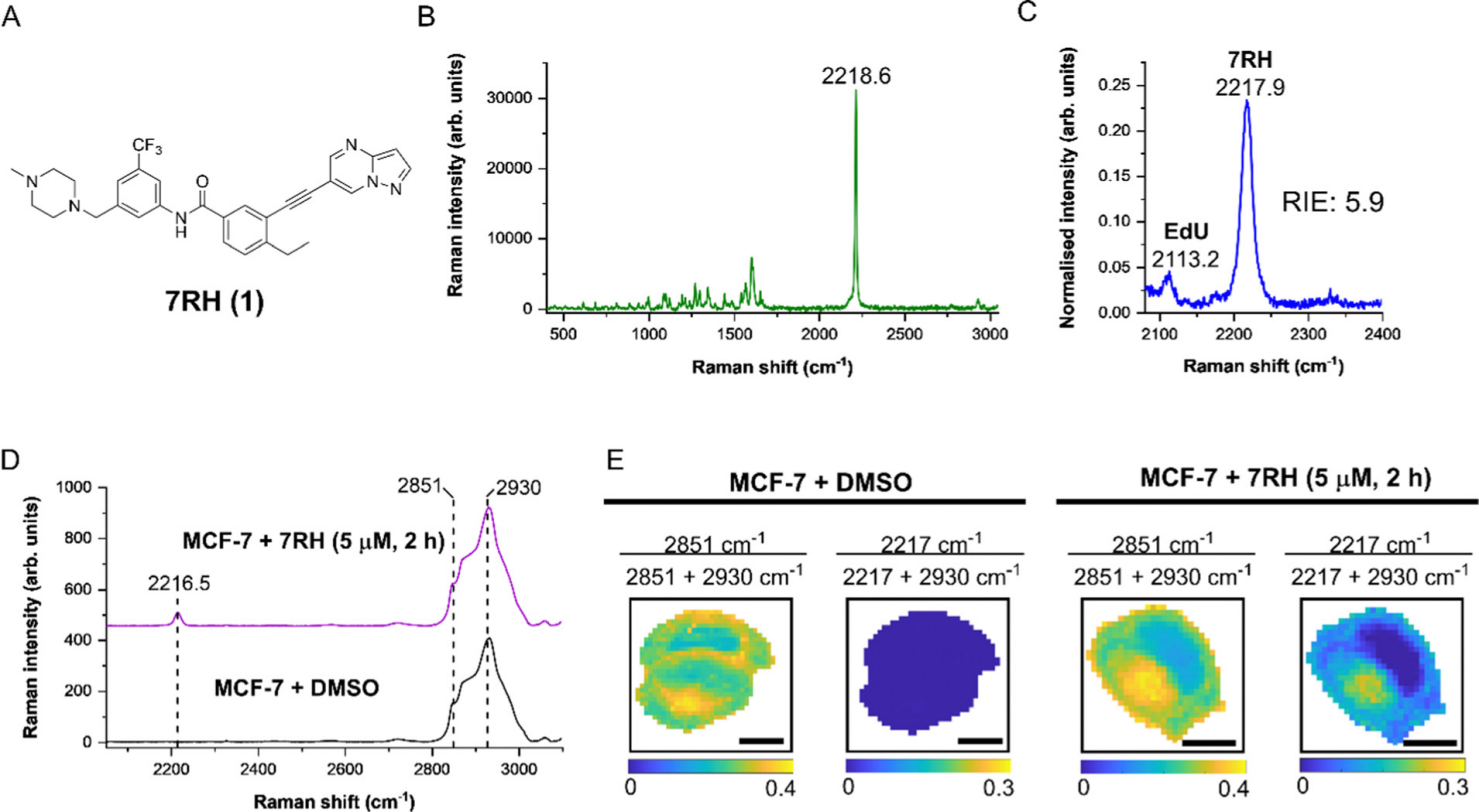
Analysis of 7RH using Raman spectroscopy. (A) Chemical structure of 7RH. (B) Raman spectrum of 7RH in solid form. Raman spectrum acquired using 532 nm excitation and a 20× lens (1.8 mW) for 10 s. The alkyne stretching frequency is highlighted at 2218.6 cm^−1^. (C) Raman spectrum of a solution of EdU (50 mM) and 7RH (50 mM) in DMSO (1 : 1 v/v). Raman spectrum acquired using 785 nm excitation and a 20× lens (98 mW) for 10 s. The alkyne stretching frequencies are highlighted for EdU (2113.2 cm^−1^) and 7RH (2217.9 cm^−1^) to enable the determination of the relative Raman signal of 7RH *vs.* EdU (RIE). (D and E) Raman microscopy of live MCF-7 cells treated with DMSO (control) or 7RH (5 μM, 2 h). Raman maps were acquired using 532 nm excitation and a 60× lens (18 mW) for 0.5 s with 1 μm pixel size. The average Raman spectrum for each cell map is presented in (D) and the ratiometric Raman images of each cell map are presented in (E). Wavenumber assignments: 2216.5 cm^−1^ (C≡C, 7RH), 2851 cm^−1^ (CH_2_ symmetric stretch), 2930 cm^−1^ (CH_3_ symmetric stretch). Scale bars: 10 μm.

Discoidin domain receptor 1 (DDR1) is a collagen-activated receptor tyrosine kinase that is expressed in epithelial cells.^1^ Collective evidence indicates that dysregulation of DDR1 is implicated in a range of disorders including cancer, atherosclerosis and fibrosis among other inflammatory diseases.^2^ Accordingly, DDR1 is an emerging molecular target for drug discovery with a diverse range of inhibitors having been reported to date.^3^ The compound 7RH (1, Fig. 1A) is a selective, orally bioavailable and highly potent DDR1 inhibitor that prevents the proliferation, invasion and migration of cancer cells *in vitro*.^4^ It is structurally related to ponatinib (Iclusig®), which is a multi-targeted tyrosine kinase inhibitor approved for use in the treatment of chronic myeloid leukaemia (CML). Ponatinib has recently been shown to accumulate in the lysosomes of CML cells within 6 hours using label-free stimulated Raman scattering (SRS) microscopy in fixedtimepoint experiments.^5^ Despite this and several other studies highlighting the advantage of direct, label-free SRS microscopy for determining intracellular drug accumulation,^6–8^ only very limited progress has been made in time-resolved SRS imaging of drug uptake and activity in living cells, notwithstanding the accordant potential in drug development.^9^ Two prominent examples of real-time SRS imaging of drug uptake include the detection of the natural product, anisomycin using a butadiyne label for high sensitivity analysis and imaging over a 1 hour timeframe,^10^ whilst the real-time detection of sucrose transport was enabled using a propargyl tagging strategy.^11^ In each case, these experiments are single-plex, where the uptake of a single alkyne labelled species was monitored in the same cell population. The sequential treatment and imaging of a cell population using both small molecule drugs and phenotypic stains has yet to be reported. For the first time, the direct washing and re-labelling of live cells with alternative probe molecules for phenotypic assessment of cellular status is reported using perfusion chamber culture and SRS microscopy.

To facilitate the detection of low-concentration species within a cellular environment, bio-orthogonal groups including alkynes, nitriles and deuterated species have been applied in SRS microscopy.^12^ These groups may be inherent to the molecule under investigation or are incorporated into the molecular structure *via* tagging.^13^ The detection of alkyne groups is desirable because they generally produce a single, sharp peak within the cell-silent region of the Raman spectrum (1800–2600 cm^−1^) thus improving specificity, whilst they generate a large Raman scattering cross section for sensitive detection.^14^ A variety of bioactive small molecules have been detected using alkyne tagging, including natural products,^10,15,16^ drugs^17,18^ and sugars^11,19,20^ with high detection sensitivity.

Herein, we describe the detection of 7RH (1), an alkyne containing DDR1 inhibitor, using SRS microscopy. We demonstrate the intracellular detection of 7RH in a variety of mammalian cell lines and investigate the uptake of 7RH in cell lines with varying expression profiles of DDR1. Furthermore, we develop a perfusion chamber culture system that enables the real-time imaging of 7RH in live cell cultures that can be imaged directly using SRS microscopy under biocompatible conditions. This system enables the recurrent treatment and imaging of live cells under physiological conditions (37 °C, 5% CO_2_ in cell culture medium) to visualise the uptake of 7RH in real-time. Following this, the viability of the cell population was assessed using fluorescent markers by means of sequential perfusion and image acquisition using a multimodal microscope system. With a perfusion protocol established, we investigated the uptake, localisation and viability of MCF-7 cells in response to 7RH and in combination with cisplatin. Finally, by capitalising on the fast-image acquisition rates associated with SRS microscopy, the effects on cellular adhesion and migration in response to 7RH treatment have been investigated. This study highlights the utility of label-free SRS microscopy for phenotypic imaging in live cell cultures which is ideally suited to the pre-clinical phase of drug development.

## Results and discussion

We first characterised 7RH using Raman microscopy, which generated an intense peak at 2218.6 cm^−1^ (C≡C, 7RH) indicative of the alkyne group (Fig. 1B). We also determined the alkyne stretching intensity of 7RH as a relative intensity to EdU (RIE), which was measured to be 5.9-fold more intense than EdU (Fig. 1C). This observation is consistent with previous RIE measurements of conjugated alkyne groups,^14^ and also indicated the potential for dual-colour imaging of 7RH (2217.9 cm^−1^) and EdU (2113.2 cm^−1^) in the same sample, given the baseline resolution of the two peaks that are separated by >100 cm^−1^.

As DDR1 is highly expressed in epithelial cells, we elected to study the effects of 7RH in MCF-7 cells, which are a model of human breast epithelial adenocarcinoma that have been shown to express high levels of DDR1.^21^ We treated MCF-7 cells with DMSO (control) or 7RH at a concentration of 5 μM for 2 h. The cells were imaged using Raman microscopy with excitation at 532 nm and a 1 μm pixel size for high resolution mapping. The average Raman spectra from representative cells are presented in Fig. 1D. The spectra were normalised to the CH_3_ stretching mode at 2930 cm^−1^, and 7RH was clearly detected at 2216.5 cm^−1^ (C≡C, 7RH) in the cell-silent region. We next performed ratiometric analysis of the Raman maps to investigate 7RH uptake and distribution (Fig. 1E). Ratiometric images were generated at 2851 cm^−1^ (CH_2_ symmetric stretch) relative to the total 2851 cm^−1^ + 2930 cm^−1^ signal. This ratio resolves the cell nucleus with a relatively lower ratio compared to the surrounding cytoplasm and lipid droplets. Intracellular 7RH was detected using the ratio 2217 cm^−1^/2217 cm^−1^ + 2930 cm^−1^ and showed that 7RH was predominantly located within the cell cytoplasm with negligible signal localised in the nucleus, which was validated by an absence of alkyne signal across the control treated cells. As such, Raman imaging revealed significant levels of 7RH accumulation within the cytoplasm of the MCF-7 cells within two hours of treatment.

**Fig. 2:**
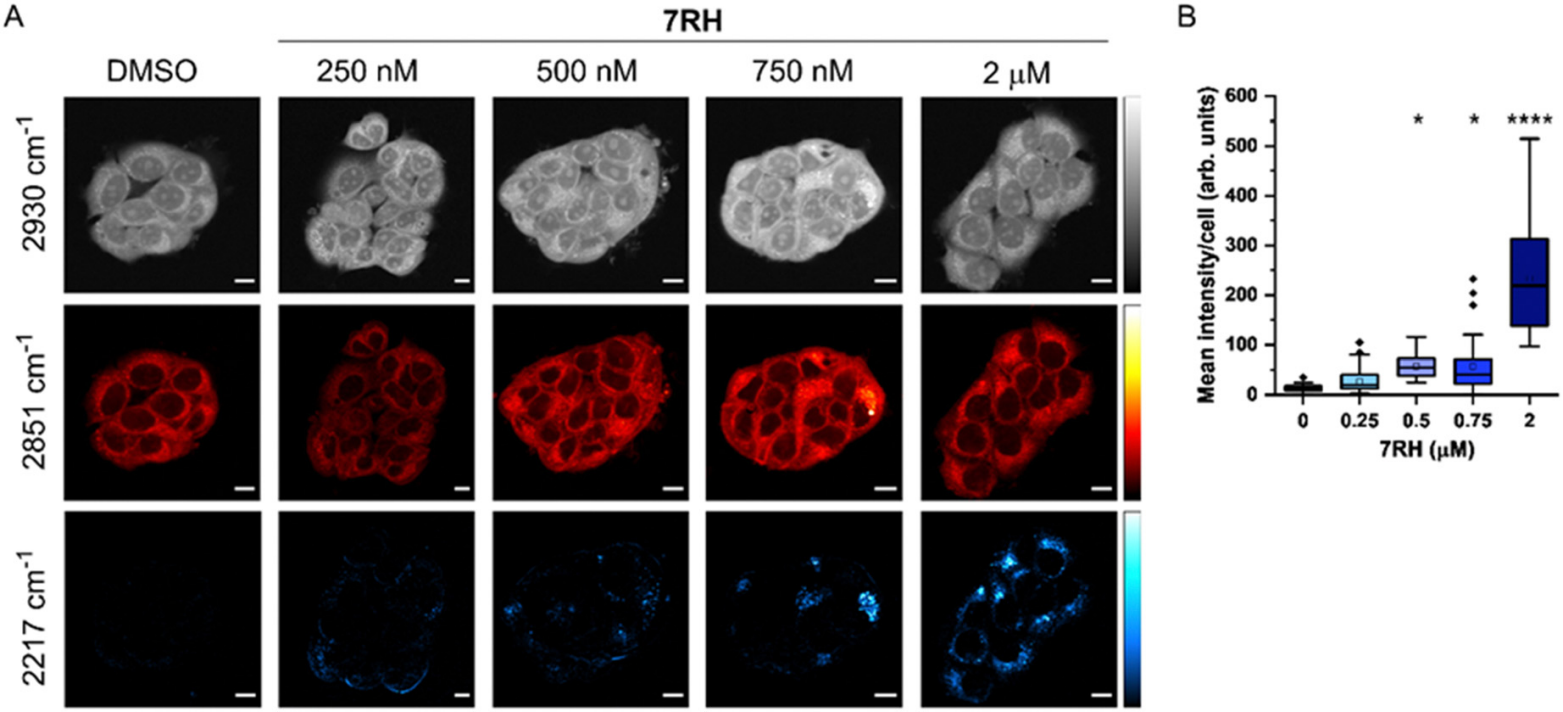
Investigating the uptake of 7RH into MCF-7 cells at different treatment concentrations. (A) MCF-7 cells were treated DMSO (control) or 7RH at the indicated concentrations for 2 h. Cells were imaged live at the following frequencies: 2930 cm^−1^ (CH_3_ symmetric stretch), 2851 cm^−1^ (CH_2_ symmetric stretch) and 2217 cm^−1^ (C≡C, 7RH). An off-resonance image was acquired at 2117 cm^−1^ which was subtracted from the 2217 cm^−1^ image. SRS images were acquired across 512 × 512 pixels, 24 μs per pixel and with false colours applied to detection wavenumbers. Scale bars: 10 μm. (B) Quantification of the mean 2217 cm^−1^ signal per cell. The mean 2217 cm^−1^ intensity per cell is quantified from *n* > 15 cells from three replicate samples. A one-way ANOVA analysis with Tukey *post hoc* analysis was performed; *P ≤ 0.05, ****P ≤ 0.0001.

SRS microscopy combines fast image acquisition with wide-area sample coverage (up to mm frame sizes) to enable a greater population coverage of cell samples and whole-tissue analysis.^22^ We next investigated the uptake of 7RH into live MCF-7 cells using SRS microscopy at fixed timepoints (Fig. 2). SRS images were acquired at 2930 cm^−1^ (CH_3_ symmetric stretch), 2851 cm^−1^ (CH_2_ symmetric stretch) and 2217 cm^−1^ (C≡C, 7RH). An off-resonance image was acquired at 2117 cm^−1^ (cell-silent region) which was subtracted from the 2217 cm^−1^ images. The images at 2930 cm^−1^ identify cellular protein and lipid signal throughout the cell cytoplasm, cell nucleus and nucleoli. Lipid droplets were detected in SRS images acquired at 2851 cm^−1^, and together, these two images provide clear label-free detection of the live cell population without contrast agents or stains. The distribution of 7RH was clearly observed in the cytoplasm of MCF-7 cells when treated with 7RH at concentrations greater than 500 nM (Fig. 2A). We quantified the intensity of alkyne signal per cell at each concentration, which confirmed significant levels of drug accumulation at concentrations greater than 500 nM at two hours of treatment (Fig. 2B). When a higher treatment concentration of 5 μM was used, the intracellular detection of 7RH could be achieved within 30 min, which continued to accumulate over 24 h (Fig. S1, ESI†).

**Fig. 3:**
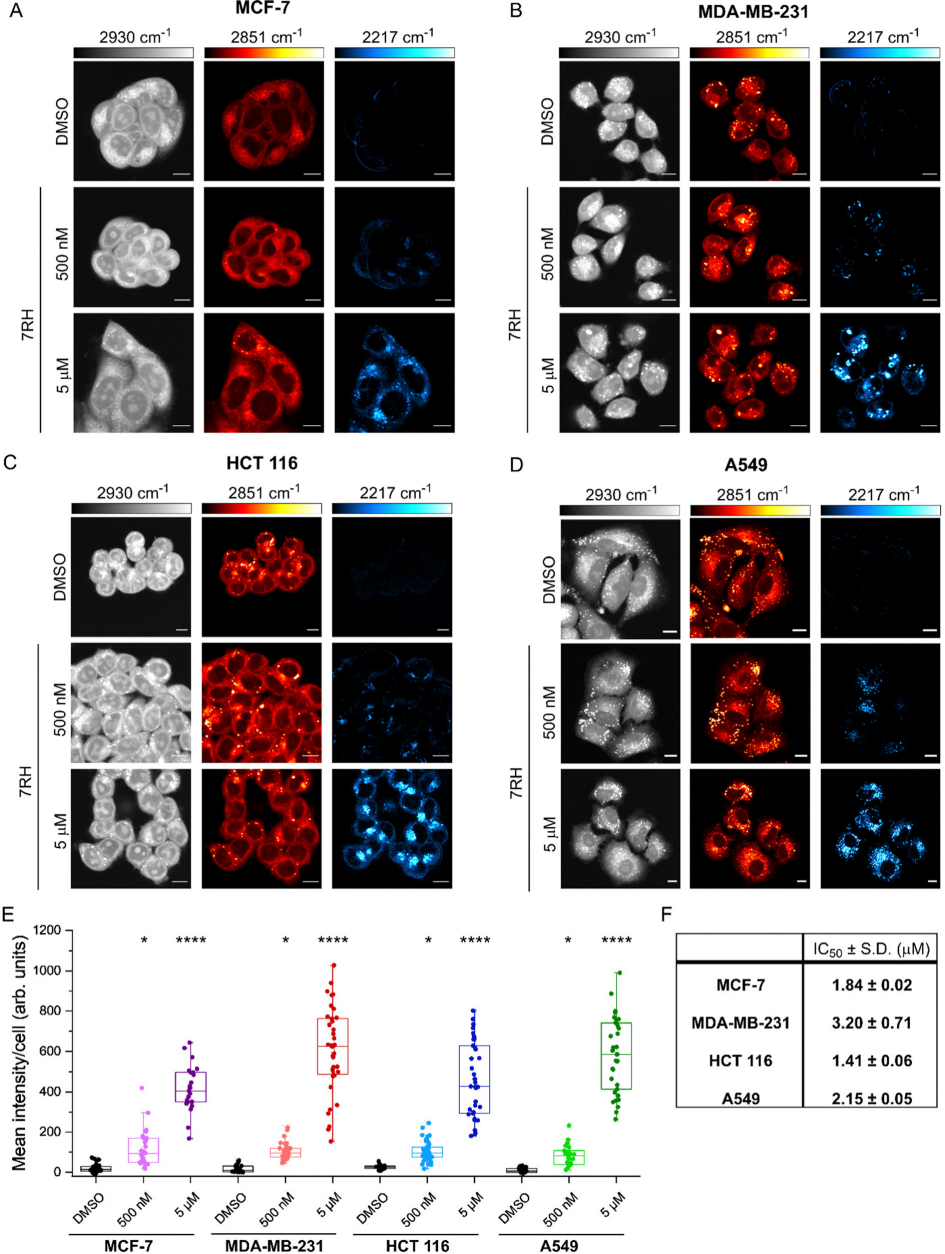
Investigating the uptake of 7RH in live cells using SRS microscopy. Live cells were treated with DMSO (control) or 7RH (500 nM or 5 μM) for 2 h. SRS images were acquired at 2930 cm^−1^ (CH_3_, proteins), 2851 cm^−1^ (CH_2_, lipids), 2217 cm^−1^ (C≡C, 7RH). An off-resonance image was acquired at 2117 cm^−1^ (cell-silent region) which was subtracted from the 2217 cm^−1^ image. SRS Images were acquired from (A) MCF-7 cells, (B) MDA-MB-231 cells, (C) HCT 116 cells and (D) A549 cells using 512 × 512 pixels, 24 μs per pixel and with false colours applied to detection wavenumbers. Scale bars: 10 μm. (E) The mean 2217 cm^−1^ intensity per cell is quantified from *n* > 25 cells in cell line. A one-way ANOVA analysis with Tukey *post hoc* analysis was performed; *P ≤ 0.05, ****P ≤ 0.0001. (F) IC_50_ ± S.D. determined in each cell line using an Alamar Blue assay, *n* = 12.

We next assessed the uptake of 7RH in a variety of cell lines with different expression profiles of DDR1: MCF-7 (breast epithelial adenocarcinoma), MDA-MB-231 (breast epithelial adenocarcinoma), HCT 116 (colorectal epithelial adenocarcinoma) and A549 (lung epithelial carcinoma). Of these, MCF-7 cells have been shown to express high levels of DDR1, whilst MDA-MB-231 cells express low levels of DDR1.^21^ We compared the uptake of 7RH in the breast cancer cell lines to the uptake in HCT 116 and A549 cancer cells that both express high levels of DDR1.^4^ In each case, cells were treated with DMSO (control) or 7RH at a concentration of 500 nM or 5 μM for 2 h, before live cell imaging using SRS microscopy. Fig. 3A–D presents the SRS microscopy data the four cell lines. SRS imaging at 2851 cm^−1^ (CH_2_ symmetric stretch) identified high levels of lipid droplets (LDs) within the MDA-MB-231 and A549 cells (Fig. 3B and D), particularly when compared to MCF-7 cells, which had very few LDs (Fig. 3A). In each case, we quantified the alkyne signal per cell (see Experimental for details), and these data showed a dose-dependent uptake in alkyne scattering in the cytoplasm of each cell type. Quantification revealed significant uptake of 7RH at 500 nM and 5 μM within 2 h in each of these cell lines (Fig. 3E). Noticeably, 7RH is largely absent from the nuclear region of the cells and is locally concentrated in puncta within the cytoplasm. In particular, MDA-MB-231 cells showed the greatest spread in the mean alkyne signal per cell at 5 μM, compared to the other three cell lines which express high levels of DDR1. Furthermore, we assessed the cytotoxicity of 7RH against each cell line (Fig. 3F), and our results identified a greater cytotoxic effect in MCF-7, HCT 116 and A549 cells which express high levels of DDR1 when compared to MDA-MB-231 cells which express a low level of DDR1. However, quantification of the alkyne signal suggested that at both concentrations tested, the uptake of 7RH appeared to be similar across the panel of cell lines tested, and as such, suggesting the uptake of the drug at these concentrations and timepoints was independent of DDR1 expression.

We further investigated the uptake of 7RH into A549 cells (Fig. S2A and B, ESI†). Significant intracellular 7RH accumulation was detected within the cells following treatment with 7RH (500 nM) after 4 h. Furthermore, we identified that 7RH is likely localised in the lysosomes of A549 cells using a multimodal imaging experiment: 7RH was detected at 2217 cm^−1^ using SRS microscopy and cellular lysosomes were stained with LysoTracker Red and detected using fluorescence microscopy (Fig. S2C, ESI†). As a weakly basic drug containing a piperazine motif (p*K*_a_ ≈ 9), the accumulation of 7RH into acidic lysosomes was anticipated and corroborates previous observations of other tyrosine kinase inhibitors including ponatinib,^5^ imatinib and nilotinib,^6^ and neratinib.^23^ Our study has identified that the uptake of DDR1 inhibitor, 7RH appears to be independent of DDR1 expression, despite toxicity being greatest in the cell lines expressing higher levels of DDR1.

We next investigated the uptake of 7RH into live MCF-7 cells using a custom designed perfusion chamber system which enabled recurrent drug treatment and imaging upon the same cell population (Fig. S3, ESI†). As identified earlier, several studies have investigated drug uptake into live cells at fixed timepoints, although limited progress has been made to assess uptake into live cells in real-time. Previous examples have used SRS microscopy and alkyne tagging strategies of natural products to enable real-time uptake analysis into mammalian cells,^10,16^ and alkyne labelling of sucrose for detection in live plant cells over 30 min.^11^ Surface-enhanced Raman scattering (SERS) has been used to study alkyne labelled drug uptake, with gold nanoparticles providing an antenna for signal amplification.^24^ However, these studies are single-plex in that only the alkyne species was imaged over time. Here, we demonstrate the uptake of unlabelled 7RH into live cell populations using SRS microscopy through the use of perfusion chambers, which for the first time enabled the direct washing and re-labelling with alternative probe molecules for phenotypic assessment of cellular status.

**Fig. 4:**
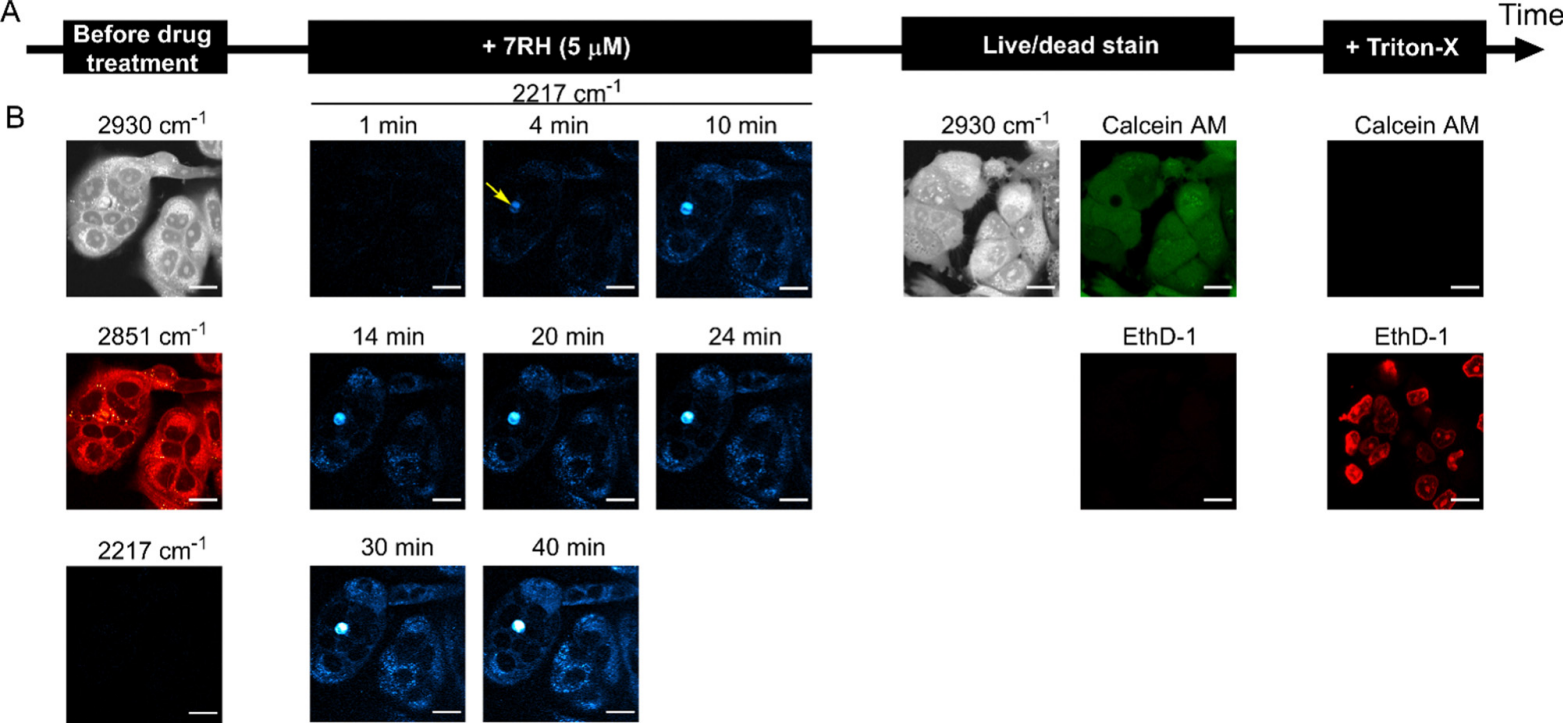
Assessing 7RH uptake into live MCF-7 cells using a perfusion chamber and multimodal imaging. (A) Schematic workflow describing cell culture under media exchange conditions. (B) SRS images were acquired at 2930 cm^−1^ (CH_3_ symmetric stretch), 2851 cm^−1^ (CH_2_ symmetric stretch), 2117 cm^−1^ (cell-silent region) and 2217 cm^−1^ (C≡C, 7RH) prior to treatment with 7RH. The cells were then treated with 7RH at 5 μM and SRS images were acquired at 2217 cm^−1^ every minute for 45 minutes. Representative images are provided at the indicated timepoints. The cells were then washed with PBS prior to imaging at 2930 cm^−1^ before staining with a solution of Calcein AM (*λ*_ex_ = 488 nm; *λ*_em_ = 495–525 nm, live cells) and ethidium homodimer-1 (EthD-1, *λ*_ex_ = 514 nm; *λ*_em_ = 535–650 nm, dead cells) in PBS. The cells were then washed with PBS before permeabilization with Triton-X and subsequent staining with Calcein AM and EthD-1. Scale bars: 20 μm.

To demonstrate the utility of this approach for assessing drug activity in a live cell population, we devised a workflow to assess the real-time uptake of 7RH before assessing the cell viability using established fluorescent staining protocols (Fig. 4A). Firstly, MCF-7 cells were seeded into the perfusion chambers and cultured for 24 h. SRS images were acquired in the high wavenumber region prior to perfusion with a solution of 7RH in culture medium. We elected to study the uptake of 7RH at 5 μM (Fig. 4B) and 1 μM (Fig. S4, ESI†); concentrations above and below the measured IC_50_ value for MCF-7 cells (1.84 μM, Fig. 3F). In each case, SRS images were acquired at 2 minute intervals from the same population of cells which were incubated under physiological conditions. Our results identified the rapid uptake of 7RH in live cells, particularly at 5 μM. It is worthy of mention that the background signal in the SRS images is still relatively low despite the treatment in complete cell culture media. After 45 mins, the cells were washed with PBS *via* perfusion, before staining with a commercial live/dead stain comprising calcein AM (live cells, 488 nm) and ethidium homodimer-1 (EthD-1, dead cells, 514 nm). Calcein AM is a cell-permeant dye that is converted to the green-fluorescent dye, calcein, upon hydrolysis of the acetoxymethyl groups by intracellular esterases in live cells, whereas EthD-1 is a high affinity nucleic acid stain that emits red fluorescence upon binding to DNA, and is impermeant to live cells, thus it identifies dead/permeabilised cells. As such, following the analysis of 7RH treatment at all concentrations, the cells were still viable as evidenced by positive calcein AM staining. This result indicated that within the timeframe of the experiment, the cells were viable, and additionally highlighted the biocompatibility of the time-lapse SRS imaging experiment. To validate the live/dead staining, we further washed the cell population with PBS before perfusing a solution of the live/dead stain containing Triton X-100 which is known to permeabilise the cell membrane. Significant EthD-1 signal was identified in the nuclei of the cells in both experiments indicating the successful perfusion of the staining solution. As such, these experiments are the first examples of sequential perfusion and imaging using SRS microscopy to study drug uptake and viability in the same cellular population using a multimodal imaging set up. Finally, we also performed a control experiment whereby time-lapse SRS imaging was performed in DMSO control treated cells (Fig. S5, ESI†). In this case, there was negligible SRS signal detected at 2217 cm^−1^ in the time course experiment, and following treatment with live/dead stain, the cells were shown to be viable, further reinforcing the biocompatibility of the SRS imaging experiment.

**Fig. 5:**
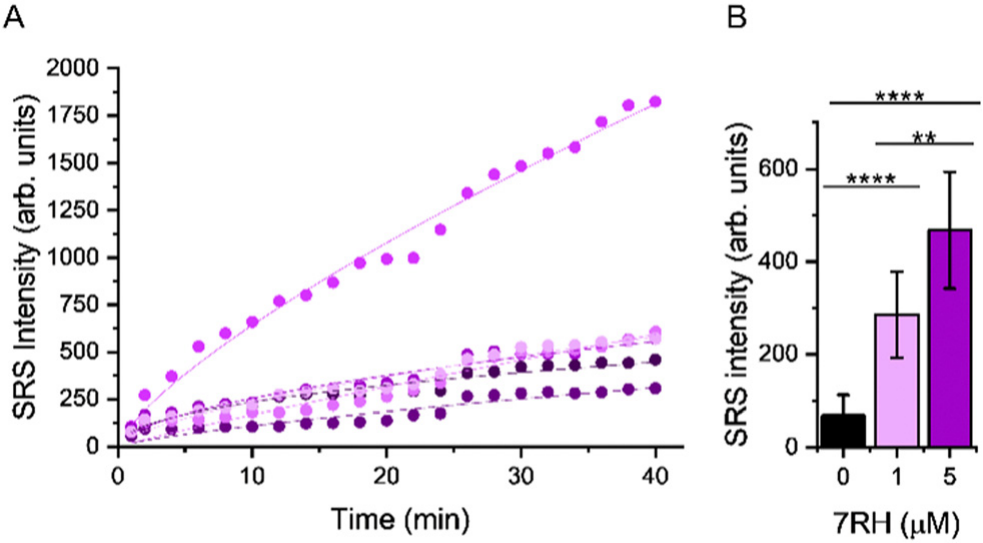
Assessing the uptake of 7RH into MCF-7 cells in a perfusion chamber. (A) Mean intensity of alkyne signal per cell following perfusion with 5 μM 7RH. A non-linear curve fitting has been applied to the uptake curve of 5 selected cells imaged in Fig. 4B. (B) Quantification of the mean alkyne signal per cell from cells treated with varying concentrations of 7RH after 40 min of treatment. Data represent the mean alkyne intensity, error bars ±S.D. A student's *t*-test was used to determine significance, **P ≤ 0.01. ****P ≤ 0.0001.

We quantified the alkyne intensity per cell following perfusion with 5 μM 7RH, which showed significant variability in the rate of uptake and overall uptake across the cells analysed after 40 min (Fig. 5A). The uptake of 7RH into one cell in the field-of-view is far greater than the others (highlighted by a yellow arrowhead, Fig. 4B); it is a rounded cell that we propose is undergoing mitosis, and appears to detach following the sequential PBS washing and staining with Calcein-AM/EthD-1 solution. Mitotic cells are known to be less firmly attached to glass substrates compared to interphase cells.^25^ Furthermore, we quantified the alkyne signal at 40 mins, and showed significant levels of accumulation at both concentrations tested (1 μM and 5 μM) when compared to the DMSO control (Fig. 5B). Interestingly, despite a 5-fold excess in drug concentration, the mean uptake at 5 μM is only 1.6-fold greater than the measured uptake when 1 μM of 7RH is used. In a following experiment, we visualised the uptake of 7RH into live MCF-7 cells before labelling with Lysotracker Green which showed colocalised signals from 7RH (2217 cm^−1^, red) and Lysotracker Green (Lyso-Green, *λ*_ex_ = 488 nm) (Fig. S6, ESI†). The piperazine unit of 7RH renders the molecule susceptible to lysosomal accumulation as previously observed for other tyrosine kinase inhibitors.^5,6^ The cells were then washed with PBS before staining with Ethidium homodimer-1; an absence of signal indicated the cells were still viable after the initial 7RH treatment. Using a perfusion chamber set-up, it is possible to image multiple intracellular targets in the same population.

**Fig. 6:**
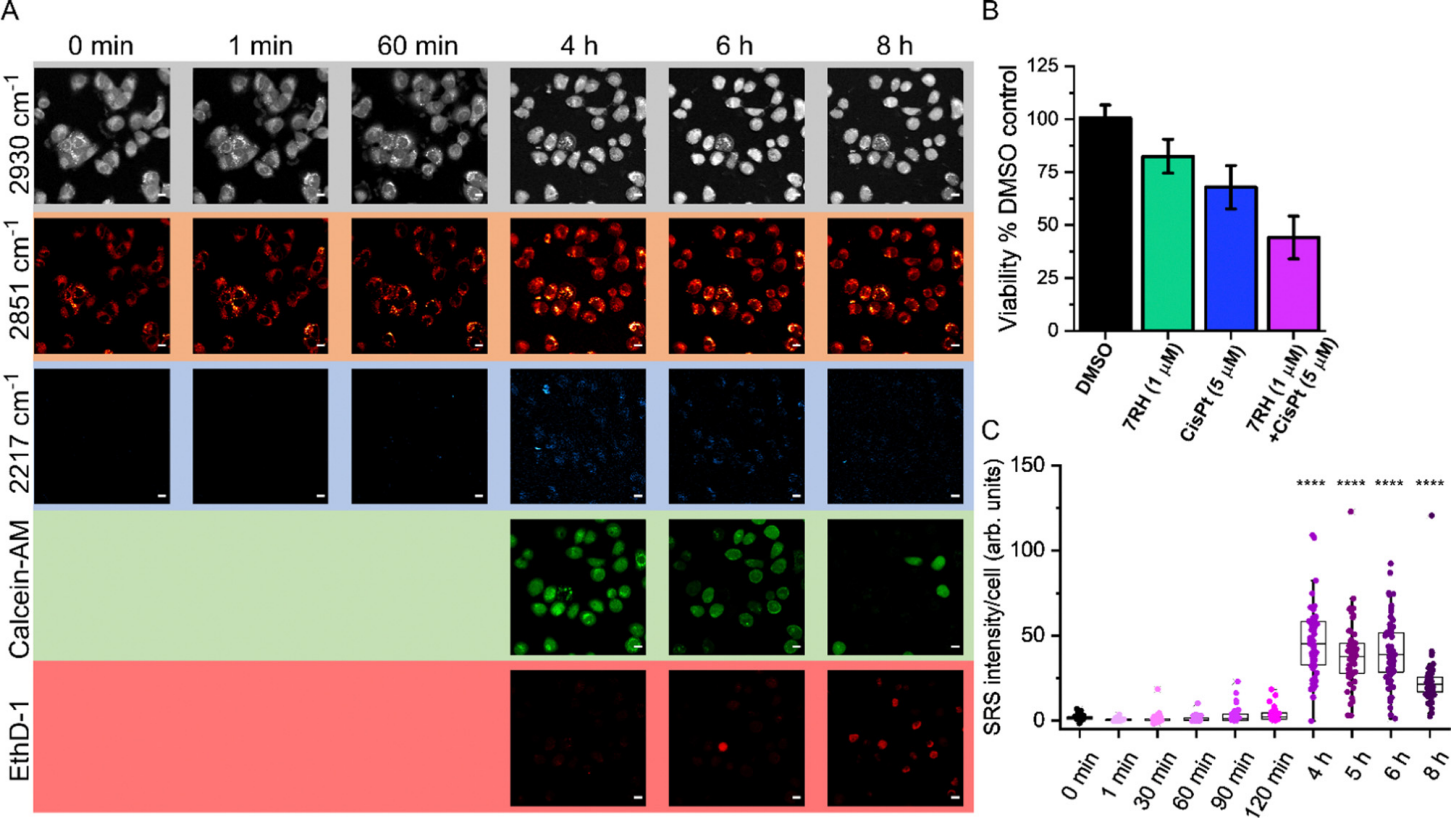
Multimodal imaging of drug activity in A549 cells using a perfusion chamber. (A) At 0 min, A549 cells were first imaged using SRS microscopy at 2930 cm^−1^, 2851 cm^−1^ and 2217 cm^−1^. The cells were then treated with 7RH (1 μM) and cisplatin (5 μM) and images were acquired at 2930 cm^−1^ and 2217 cm^−1^ at the indicated timepoints (1 min onwards). After the 4 h timepoint, the cells were stained with a solution of Calcein-AM and Ethdium homodimer-1 (EthD-1). SRS images were acquired at 2930 cm^−1^, 2851 cm^−1^ and 2217 cm^−1^, and fluorescence microscopy (Calcein-AM, *λ*_ex_ = 488 nm; EthD-1, *λ*_ex_ = 514 nm) up to 8 h. Scale bars: 10 μm. (B) Analysis of A549 cell viability using Alamar Blue assay. A549 cells were treated with (i) DMSO, (ii) 7RH (1 μM), (iii) cisplatin (5 μM) or (iv) 7RH (1 μM) and cisplatin (5 μM) for 72 h. Cell viability expressed as a percentage of the mean value in each case with error bars: ±S.D. *n* = 12. (C) Quantification of the alkyne signal intensity at 2217 cm^−1^ per cell (in (A) *n* > 20 cells) as a function of time compared to the DMSO control. A one-way ANOVA analysis with Tukey *post hoc* analysis was performed; ****P ≤ 0.0001.

Having demonstrated the utility of a perfusion chamber system for assessing 7RH uptake and subsequent viability assessment, we next assessed the sequential treatment of live A549 cells with 7RH and cisplatin (Fig. 6). The use of combination therapies is an important therapeutic strategy in minimising drug resistance.^26^ We elected to study the activity of 7RH and cisplatin which is a potential combination therapy for lung cancer treatment.^27^ In this experiment, the uptake of 7RH (1 μM) into live cells in co-treatment with cisplatin (5 μM) was assessed from the same cell population over 8 h. SRS images were acquired at 2930 cm^−1^ (CH_3_), 2851 cm^−1^ (CH_2_) and 2217 cm^−1^ (C≡C, 7RH) in a live cell population maintained at 37 °C and 5% CO_2_ atmosphere. After 4 h treatment time, the cells were also co-stained with calcein-AM (live cells, *λ*_ex_ = 488 nm) and ethidium homodimer-1 (EthD-1; dead cells, *λ*_ex_ = 514 nm). Interestingly, the cells were viable for up to 6 h under these conditions, with very weak signal detected in the EthD-1 images. The intensity of the EthD-1 signal begins to increase after 6 h with a corresponding reduction in calcein-AM positive cells, indicating an overall reduction in the viability of the cellular population. The SRS images at 2930 cm^−1^ and 2851 cm^−1^ identified a rounded morphology with few cell–cell contacts consistent with cell death. The combination treatment produced a greater reduction in cell viability compared to the DMSO (control) and 7RH alone (1 μM) treatments (Fig. 6B). In addition, the intensity of the signal at 2217 cm^−1^ (7RH) appears to increase over the course of the experiment, although a reduction of 7RH signal was detected after 6 h (Fig. 6C). At 8 h, there was an apparent reduction in the overall 7RH intensity per cell which was likely due to a reduction in cell viability as identified by the increase in EthD-1 positive cells. This experiment highlights the clear potential of live-cell perfusion imaging for multiplex analysis of drug–cell interactions using a multimodal imaging platform over an extended period. As such, a greater insight into drug–cell interactions can be visualised across the same cellular population using a wide variety of Raman and fluorescent labels.

**Fig. 7:**
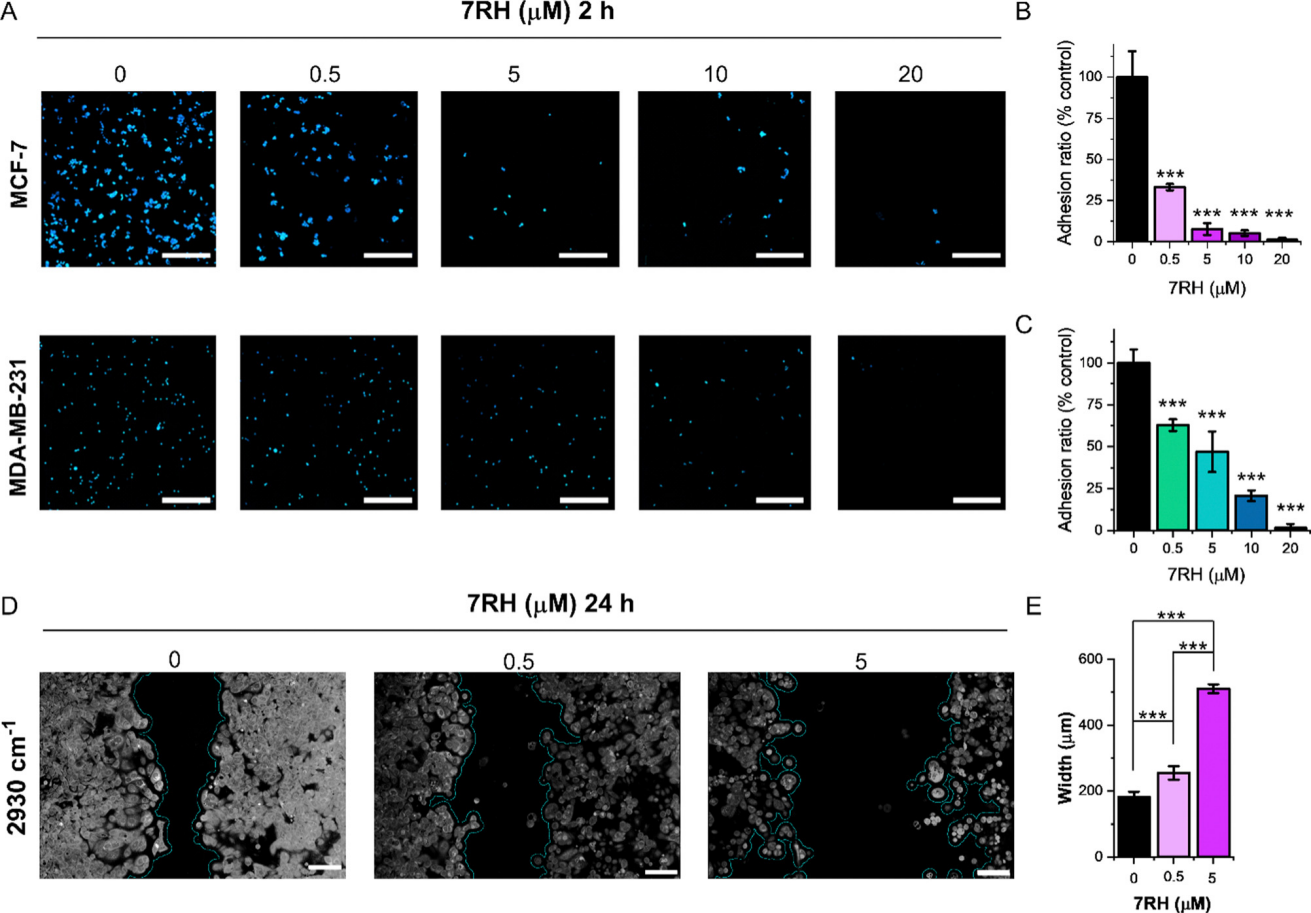
Investigating the effects of 7RH on cellular adhesion and migration using SRS microscopy. (A) MCF-7 or MDA-MB-231 cells were plated directly onto glass coverslips in the presence of 7RH at the indicated concentrations for 2 h. SRS images were acquired at 2930 cm^−1^ (CH_3_, proteins) across a 1.4 mm frame from the coverslip (*n* = 6). Scale bars: 350 μm. (B) Quantification of adhesion ratio of MCF-7 cells as a function of 7RH concentration. (C) Quantification of adhesion ratio of MDA-MB-231 cells as a function of 7RH concentration. A Student's *t*-test was used to compare the adhesion ratio for the 7RH treatments to the DMSO control; ***P ≤ 0.001. (D) Analysis of cellular migration using a scratch-wound assay. MCF-7 cells were grown to confluency in two chambers separated by 500 μm, after which the culture insert was removed, and the cells treated with 7RH at the indicated concentrations for 24 h. Wide-area SRS imaging was performed at 2930 cm^−1^ (CH_3_, proteins) across the scratch-wounds at each condition. Scale bars: 100 μm. (E) Quantification of the width of the scratch at each concentration. A Student's *t*-test was used to compare the gap width for the 7RH treatments to the DMSO control; ***P ≤ 0.001.

Finally, we demonstrated the application of SRS microscopy for cell culture-based phenotypic screening. Cellular adhesion mechanisms underpin the organisation of cells in tissue architectures. DDR1 is implicated as a key regulator of cell adhesion, and the impact of 7RH inhibition on cellular adhesion is therefore of crucial importance.^28^ We assessed the adhesion of MCF-7 (DDR1 high) and MDA-MB-231 (DDR1 low) cells using an SRS imaging approach. In each case, cells were plated at a density of 0.5 million per mL in culture medium containing 7RH (0.5–20 μM) or an equivalent volume of DMSO as a control. After 2 h incubation, the slides were washed to remove unattached cells, and fixed using paraformaldehyde. To improve the reliability of the cell counting, we acquired SRS images across an area of 1.4 × 1.4 mm by stitching images together in a 4 × 4 grid with individual frame size of 350 × 350 μm. As the concentration of 7RH increases, there is a significant reduction in the adhesion of both the MCF-7 and MDA-MB-231 cell lines to the glass substrate (Fig. 7). In each case, treatment with 7RH at concentrations greater than 5 μM, resulted in a >50% reduction in cellular adhesion when compared to the DMSO control (Fig. 7B and C). Inhibition of adhesion appears to be greatest in the MCF-7 (DDR1 high) cells compared to the MDA-MB-231 (DDR1 low) cells, which follows a similar trend observed in the toxicity data (Fig. 3F).

Cellular migration defines the movement of cells from one location to another and is a central process in cell growth and proliferation. To study cellular migration *in vitro*, the scratch-wound assay has been developed, whereby a gap is created within a confluent monolayer of cells, and the proliferating edge of cells will migrate into the newly created gap.^29^ To assess the effects of 7RH upon cell migration, MCF-7 cells were cultured for 48 h using an insert comprising two chambers separated by 500 μm. The inserts were removed to allow proliferating cells to migrate into the 500 μm gap that had been created, and the cells were cultured for a further 24 h in the presence of 7RH (0.5 and 5 μM) or DMSO (control). We analysed the cultures using SRS imaging across a frame size of 1.1 × 0.7 mm and determined the width of the gap (Fig. 7D). Our results indicated a dose dependency in the extent of the closure; control treated cells reduced the gap to a mean value of ∼180 μm, whilst cells treated with 7RH at a concentration of 0.5 μM resulted in a gap closure of ∼250 μm, and cells treated with 7RH at a concentration of 5 μM, resulted in no gap closure with a noticeable reduction in cell coverage as a further indicator of toxicity. We have therefore demonstrated that 7RH inhibited MCF-7 cell migration in a dose-dependent manner using a scratch-wound assay and SRS imaging readout. The analysis of the gap width was easily achieved using ImageJ; a threshold analysis was applied to the SRS images and the gap width was automatically calculated using a bespoke plugin,^30^ which showed a significant effect of 7RH treatment upon cellular migration (Fig. 7E). These data suggest there is significant potential for SRS microscopy to assess the cellular response to drug treatment within the preclinical drug development process.

## Conclusions

This study highlighted the application of perfusion-based experiments for recurrent dosing and imaging of a live cell population to monitor drug uptake, localisation and subsequent viability assessment using commercial fluorescent stains. Whereas many previous drug uptake studies have used single, fixed timepoints in culture dishes or used coverslips mounted using imaging spacers, our study demonstrates a clear advantage through the use of perfusion chambers within environment-controlled conditions, to better replicate the physiological state. Our experiments show it is possible to detect drug uptake over a period of 8 h in living cells, whilst also assessing the cell viability and drug localisation in a multimodal imaging experiment. Furthermore, the detection of 7RH alkyne is achieved directly without the use of adding nanoparticle sensors to the cells to enhance the detection through the SERS effect. As such, the sequential treatment and imaging regime demonstrated in this work was enabled by multimodal microscopy to study drug uptake with temporal and subcellular spatial resolution, together with a viability assessment directly in the same cell population. Here, we presented the detection of three different labelling experiments in the same live cell population within 8 h under physiological conditions. The pharmacokinetics of a candidate drug is a critical parameter in the early stages of drug development, and the complex interplay between a drug and the patient render a significant challenge to understand their intracellular dynamics. We aim to investigate drug treatments to live cells over a greater range of timepoints and treatment concentrations to better replicate the bioavailability profile of a drug *in vivo*. In addition, the application of hyperspectral SRS microscopy and spectral phasor analysis has recently enabled the phenotypic assessment of drug–cell interactions,^31^ whilst the vast array of fluorescent and Raman-based sensors for cellular characterisation could be implemented into this workflow to expand the diversity of imaging experiments.

## Experimental

### Reagents and chemicals

DDR1 inhibitor, 7RH (≥98%) was purchased from Merck and used as supplied and prepared as 10 mM stock solution in anhydrous DMSO.

### Cell culture

MCF-7 cells (ATCC® HTB-22™) were obtained from American Type Culture Collection (ATCC). MDA-MB-231 cells (ATCC® HTB-26™) were gifted from the Strathclyde Institute of Pharmacy and Biomedical Sciences (Glasgow) as a subculture from a stock received from the European Collection of Authenticated Cell Cultures (ECACC). A549 cells (ECACC catalogue no. 86012804) were obtained from the ECACC. HCT 116 cells (CCL-247™) were gifted from The University of Edinburgh. MCF-7 and MDA-MB-231 cell lines were cultured in Rosewell Park Memorial Institute medium (RPMI 1640; GIBCO™, Fisher Scientific) supplemented with 10% foetal bovine serum (FBS, Gibco™, Fisher Scientific), 1% penicillin/streptomycin (Gibco™, 10 000 U mL^−1^, Fisher Scientific) and 1% Amphotericin B (Gibco™, 250 μg mL^−1^, Fisher Scientific). A549 cells were cultured in Dulbecco's Modified Eagle's Medium (DMEM low glucose, 1 g L^−1^) supplemented with 10% foetal bovine serum (FBS, Gibco™, Fisher Scientific), 1% penicillin/streptomycin (Gibco™, 10 000 U mL^−1^, Fisher Scientific) and 1% Amphotericin B (Gibco™, 250 μg mL^−1^, Fisher Scientific). HCT 116 cells were cultured in Dulbecco's Modified Eagle's Medium (DMEM high glucose, 4.5 g L^−1^) supplemented with 10% foetal bovine serum (FBS, Gibco™, Fisher Scientific), 1% penicillin/streptomycin (Gibco™, 10 000 U mL^−1^, Fisher Scientific) and 1% Amphotericin B (Gibco™, 250 μg mL^−1^, Fisher Scientific). All cells were maintained at 37 °C and 5% CO_2_ in a humidified incubator and were routinely sub-cultured at *ca.* 80% confluency.

### Raman microscopy

All Raman spectra were acquired on a Renishaw inVia Raman microscope equipped with a 532 nm Nd:YAG laser providing a maximum output of 50 mW and using a 1800 lines per mm grating. Prior to spectral acquisitions, the instrument was calibrated using the internal silicon standard at 520.5 cm^−1^. Live-cell Raman imaging: MCF-7 cells were plated on glass bottomed culture dishes (35 mm high, Ibidi) at a concentration of 2.5 × 10^5^ cells per mL and incubated at 37 °C and 5% CO_2_ for 24 h prior to treatment. Cells were treated with 7RH from 10 mM stock solution in DMSO (or DMSO as a control) and incubated at 37 °C and 5% CO_2_ for the indicated time. Prior to imaging, the dishes were aspirated and washed with PBS (2 × 2 mL) and imaged in PBS. Raman maps were acquired using *λ*_ex_ = 532 nm with a Nikon 60×, N.A. 1.0 NIR Apo water immersion objective, 1 μm step size in *x* and *y*, 0.5 s acquisition time, 50% laser power (*ca.* 18 mW) and a spectral centre of 2800 cm^−1^. Three replicate maps of different cells were acquired from a single culture plate for each condition. Ratiometric images were prepared using a custom script on MATLAB as reported previously.^32^

### SRS microscopy

An integrated laser system (picoEmerald™ S, Applied Physics & Electronics, Inc.) was used to produce two synchronised laser beams at 80 MHz repetition rate. A fundamental Stokes beam (1031.4 nm, 2 ps pulse width) was intensity modulated by an electro-optic-modulator with >90% modulation depth, and a tunable pump beam (700–960 nm, 2 ps pulse width, <1 nm (10 cm^−1^) spectral bandwidth) was produced by a built-in optical parametric oscillator. The pump and Stokes beams were spatially and temporally overlapped using two dichroic mirrors and a delay stage inside the laser system and coupled into an inverted laser-scanning microscope (Leica TCS SP8, Leica Microsystems) with optimised near-IR throughput. SRS images were acquired using 40× objective (HC PL IRAPO 40×, N.A. 1.10 water immersion lens) with a 9.75–48 μs pixel dwell time over a 512 × 512 or a 1024 × 1024 frame. The Stokes beam was modulated with a 20 MHz EoM. Forward scattered light was collected by a S1 N.A. 1.4 condenser lens (Leica Microsystems). For the analysis of scratch-wounds assays, a S1 N.A. 0.9 air condenser lens (Leica Microsystems) was used. Images were acquired at 12-bit image depth. The laser powers measured after the objective lens were in the range 10–30 mW for the pump beam only, 10–50 mW for the Stokes beam only and 20–70 mW (pump and Stokes beams).

#### Perfusion SRS measurements

Grace Bio-Labs CoverWell™ perfusion chambers (width: 3 mm, length: 32 mm, depth: 0.6 mm) were affixed onto a glass coverslip (#1.5H thickness, 25.5 mm × 75.5 mm, Ibidi) prior to cell seeding. MCF-7 cells or A549 cells were seeded at a concentration of 1 × 10^6^ cell per mL (∼80 μL) in RPMI (MCF-7) or DMEM (A549) and incubated at 37 °C and 5% CO_2_ for 24 h prior to analysis. The channels were flushed with media (∼100 μL) prior to drug treatment. The SRS microscope used for this work is housed in a climate-controlled environment where the apparatus is heated and maintained at 37 °C (Fig. S3, ESI†). In addition, the system is equipped with a CO_2_ infuser which is coupled to a humidifier which is held in the headspace above the cell culture treatment media (that is held under a positive pressure of CO_2_ and equilibrated within the microscope enclosure in a 15 mL or 50 mL Falcon tube). When required, the media was withdrawn from the Falcon tube using a syringe (1 mL, Injet Braun) and was perfused across the cells by slowly releasing the syringe contents onto one port of the perfusion chamber, whilst a second syringe was used to remove the eluent at the opposite port. During the experiment, the equipment was held at 37 °C and 5% CO_2_*via* humidifier; the perfusion chambers comprise of a gas-permeable membrane on the top. All drug treatments were performed when the perfusion chamber was in-focus on the microscope stage at the equilibrated conditions.

For drug treatment perfusion studies, approximately 200 μL solution of 7RH (1 μM or 5 μM in media) or DMSO (0.001% in media v/v) was perfused over the cell population (in approx. 1 min). SRS images were acquired at 2217 cm^−1^ at 2 minute intervals thereafter. After 40 minutes, the cells were washed by perfusing PBS (∼200 μL), before staining with live/dead fluorescent stain (ThermoFisher Scientific). In the first instance, ∼200 μL live/dead stain (2 μM Calcein AM, 4 μM ethidium homodimer-1 in PBS) was perfused across the cell population (approx. 1 minute), and after 5 minutes, fluorescence images were acquired: Calcein AM (*λ*_ex_ = 488 nm; *λ*_em_ = 495–525 nm, live cells) and ethidium Homodimer (EthD-1, *λ*_ex_ = 514 nm; *λ*_em_ = 535–650 nm, dead cells). Following this, the cells were washed by perfusing PBS (∼200 μL), before staining with live/dead fluorescent stain (2 μM Calcein AM, 4 μM ethidium homodimer-1 and 0.05% Triton-X 100 v/v in PBS) was perfused across the cell population (approx. 1 minute), and after 10 minutes, fluorescence images were acquired as before.

### Live-cell SRS imaging

Live cells were plated onto high precision glass coverslips (#1.5H thickness, 22 × 22 mm, Thorlabs) in a 6-well plate in their respective media at a concentration of 0.5 million per mL. The cells were cultured for 24 h before treatment with 7RH or DMSO (0.1% v/v control) for the indicated timepoints. The cells were washed with PBS prior to mounting onto a glass microscope slide with a boundary of PBS as described in ref. 6.

### Adhesion assay

MCF-7 or MDA-MB-231 cells were plated onto high precision glass coverslips (#1.5H thickness, 22 × 22 mm, Thorlabs) in a 6-well plate in RPMI at a concentration of 0.5 million per mL in culture medium containing 7RH (0.5–20 μM) or an equivalent volume of DMSO as a control. After 2 h incubation, the slides were washed to remove unattached cells, and fixed using paraformaldehyde (4% in PBS, 15 min at rt). The slides were mounted onto microscope slides as above.

### Scratch-wound assay

A silicone culture-insert 2-well (Ibidi) was affixed onto the base of a 35 mm glass-bottomed culture dish (Ibidi). MCF-7 cells (70 000) were seeded into each of the two chambers and cultured for 48 h with a media change after 24 h. After 48 h, the insert was removed with tweezers, and the imaging dish filled with RPMI + 7RH (5 μM or 500 nM) or DMSO (0.1% v/v). The cells were incubated for a further 24 h, before washing with PBS, and fixation with paraformaldehyde (4% in PBS, 15 min at rt). The cells were washed with PBS (2 mL) and imaged in a thin film of PBS using an S1 N.A. 0.9 air condenser lens (Leica Microsystems).

### Cell viability assessment

AlamarBlue assay: MCF-7, MDA-MB-231, A549 and HCT 116 cells were seeded into 96-well plates in respective media at a concentration of 5 × 10^4^ cells per mL and incubated at 37 °C and 5% CO_2_ for 48 h prior to treatment. The media was removed by gentle aspiration prior to treatment with either DMSO (0.025% v/v negative control), Triton X-100 (0.001% v/v positive control) or 7RH (30–0.003 μM) for 72 h. After 72 h, AlamarBlue reagent (10 μL) was added to each well and the cells incubated for 4 h, after which the fluorescence emission from each well was measured using a Tecan Spark multimode plate reader with *λ*_ex_ = 545 nm (bandwidth 15 nm) and *λ*_em_ = 590 nm (bandwidth 5 nm). The IC_50_ was determined using a sigmodal fitting on the dose-response curve and plotted using Origin2018 software.

### Data processing

#### SRS images

False colour assignments, scale bars and image overlays were added to images using ImageJ software. Consistent brightness and contrast settings were used when comparing image datasets. Images of 7RH distribution are presented by subtracting the background signal from the on-resonant signal (2217–2117 cm^−1^) using the *Image Calculator* function available on ImageJ. The mean SRS intensity of 7RH signal per cell was determined by first manually selecting individual cells based on the 2930 cm^−1^ image using the freehand selection tool (to create individual ROIs) and the intensity of the alkyne signal determined in the cellular regions (each ROI) was determined using the Analysis tool on ImageJ.

#### Scratch wound assays

SRS images were acquired across a 3 × 2 frame (350 μm × 350 μm frame size) and a merged image created using LASX software that controls the SRS microscope. The merged images were imported into ImageJ and the mean gap width determined using the Wound_Healing_Size_Tool that has been reported previously and is available from ref. 30.

## Author contributions

W. J. T., A. S. M. and R. F. conducted all experiments and analysed the results. W. J. T., N. C. O. T., K. F. and D. G. drafted the manuscript, supervised the project and are responsible for funding.

## Conflicts of interest

There are no conflicts to declare.

## Supplementary Material

CB-003-D2CB00160H-s001

## Data Availability

The research data associated with this paper will become available from the University of Strathclyde at the following link: https://doi.org/10.15129/1cd812d2-81ba-4894-8ba6-6da2acd94699.
