# Supplementary material for: Temporal imaging of drug dynamics in live cells using stimulated Raman scattering microscopy and a perfusion cell culture system
Source: RSC Chem Biol. 2022 Aug 9;3(9):1154–64. doi: 10.1039/d2cb00160h (PMC9428671; doi:10.1039/d2cb00160h)
Supplement: CB-003-D2CB00160H-s001 [file CB-003-D2CB00160H-s001.pdf]

Electronic Supplementary Material (ESI) for RSC Chemical Biology.
This journal is © The Royal Society of Chemistry 2022

Temporal imaging of drug dynamics in live cells using stimulated Raman scattering microscopy and a perfusion cell culture system

Electronic Supplementary Information

William J. Tipping,[a] Andrew S. Merchant,[a] Rebecca Fearon,[a] Nicholas C. O. Tomkinson,*[b] Karen Faulds,*[a] and Duncan Graham*[a]

a. Centre for Molecular Nanometrology, WestCHEM, Department of Pure and Applied Chemistry, Technology and Innovation Centre, University of Strathclyde, Glasgow G1 1RD, UK.
E-mail: karen.faulds@strath.ac.uk; duncan.graham@strath.ac.uk.

b. Department of Pure and Applied Chemistry, University of Strathclyde, Glasgow G1 1XL, UK.
E-mail: nicholas.tomkinson@strath.ac.uk

Contents

Material and Methods……………………………………………………………………………2

Figures S1-S6………………………………………………………………………………..3

Materials and Methods

Fluorescence imaging

A549 cells were treated with 7RH (500 nM, 2 h) before treating with lysotracker red (75 nM, 30 min). The cells were mounted in PBS containing lysotracker red (75 nM) and affixed onto a microscope slide as performed for live cell SRS imaging. Fluorescence images were acquired using a Leica Microsystems SP8 confocal microscope equipped with a 40× NA 1.10 water immersion objective lens. Lysotracker red signal was detected using λ_{ex} = 561 nm; λ_{em} = 565-650 nm (Figure S2). Pearson correlation coefficient (Figure S6) determined using Coloc2 tool on ImageJ.

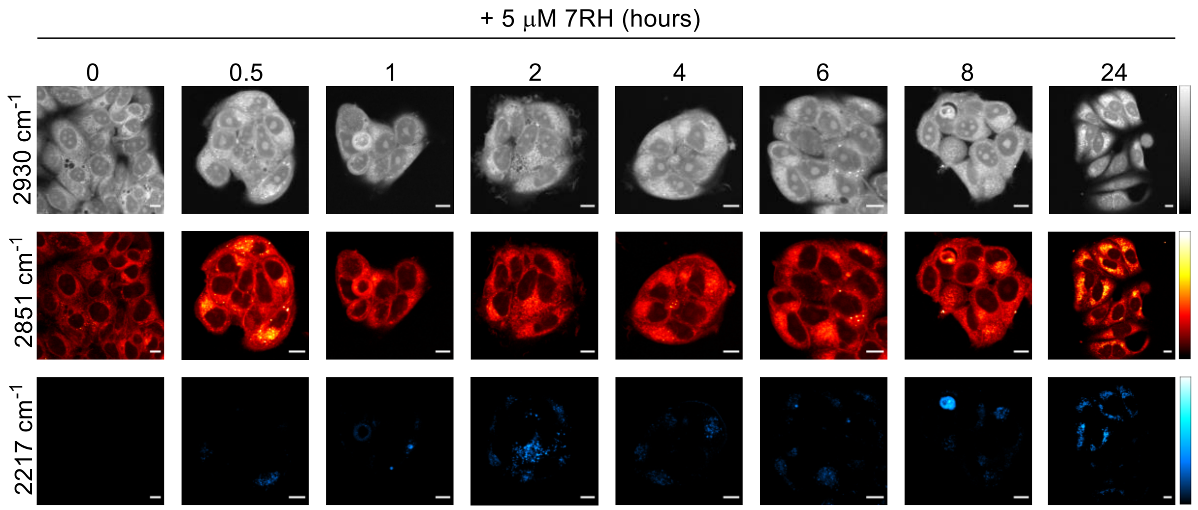

Figure S1 Investigating the uptake of 7RH into MCF-7 cells. **A** MCF-7 cells were treated with 7RH at a concentration of 5 µM for the indicated timepoints. Cells were imaged live at the following frequencies: 2930 cm^{-1} (CH_3 symmetric stretch), 2851 cm^{-1} (CH_2 symmetric stretch) and 2217 cm^{-1} (C≡C, 7RH). An off-resonance image was acquired at 2117 cm^{-1} which was subtracted from the 2217 cm^{-1} image. SRS images were acquired across 512×512 pixels, 24 µs/pixel and with false colours applied to detection wavenumbers. Scale bars: 10 µm.

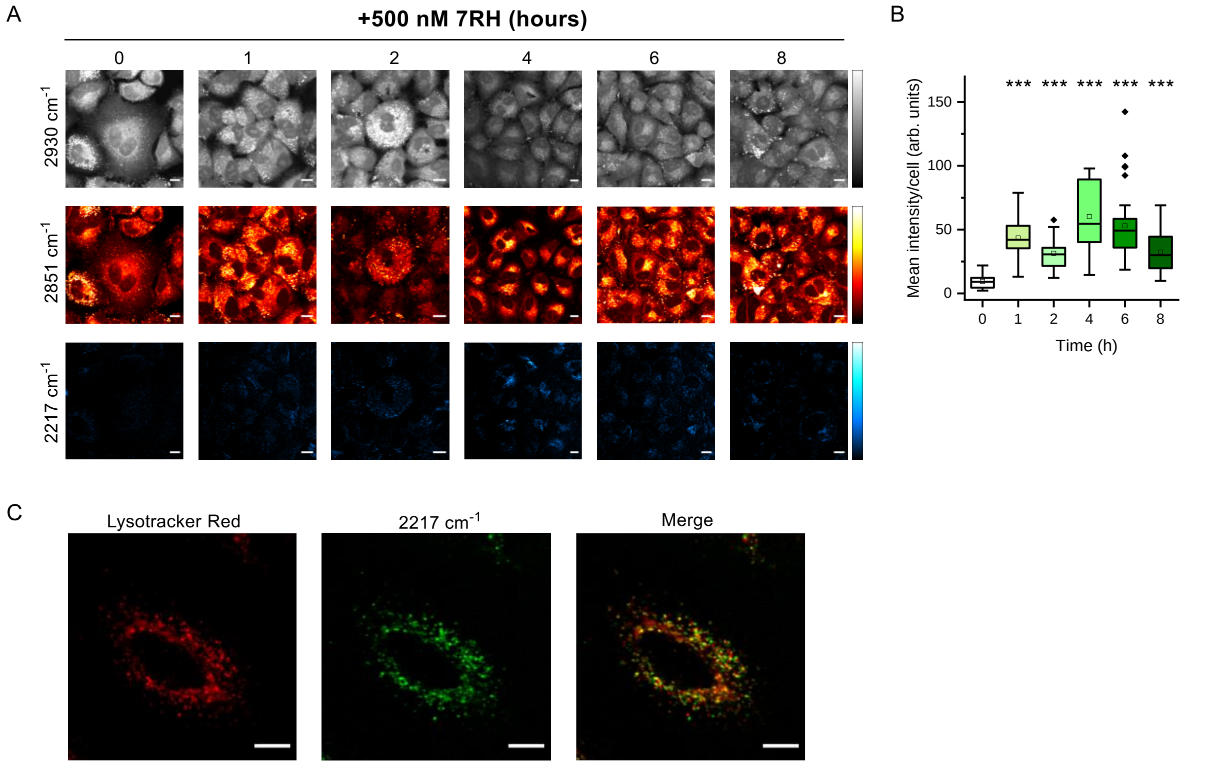

Figure S2 Investigating the uptake of 7RH into A549 cells. **A** A549 cells were treated DMSO (control) or 7RH at 500 nM for the indicated timepoints. Cells were imaged live at the following frequencies: 2930 cm^{-1} (CH_3 symmetric stretch), 2851 cm^{-1} (CH_2 symmetric stretch) and 2217 cm^{-1} (C≡C, 7RH). An off-resonance image was acquired at 2117 cm^{-1} which was subtracted from the 2217 cm^{-1} image. SRS images were acquired across 512×512 pixels, 24 µs/pixel and with false colours applied to detection wavenumbers. Scale bars: 10 µm. **B** Quantification of the mean 2217 cm^{-1} signal per cell in **A**. The mean 2217 cm^{-1} intensity per cell is quantified from n>15 cells from three replicate samples. A one-way ANOVA analysis with Tukey post-hoc analysis was performed; ***P≤0.005. **C** A549 cells were treated with 7RH (500 nM, 2h) before treating with lysotracker red (75 nM, 30 min) and were imaged live. Scale bars 10 µm.

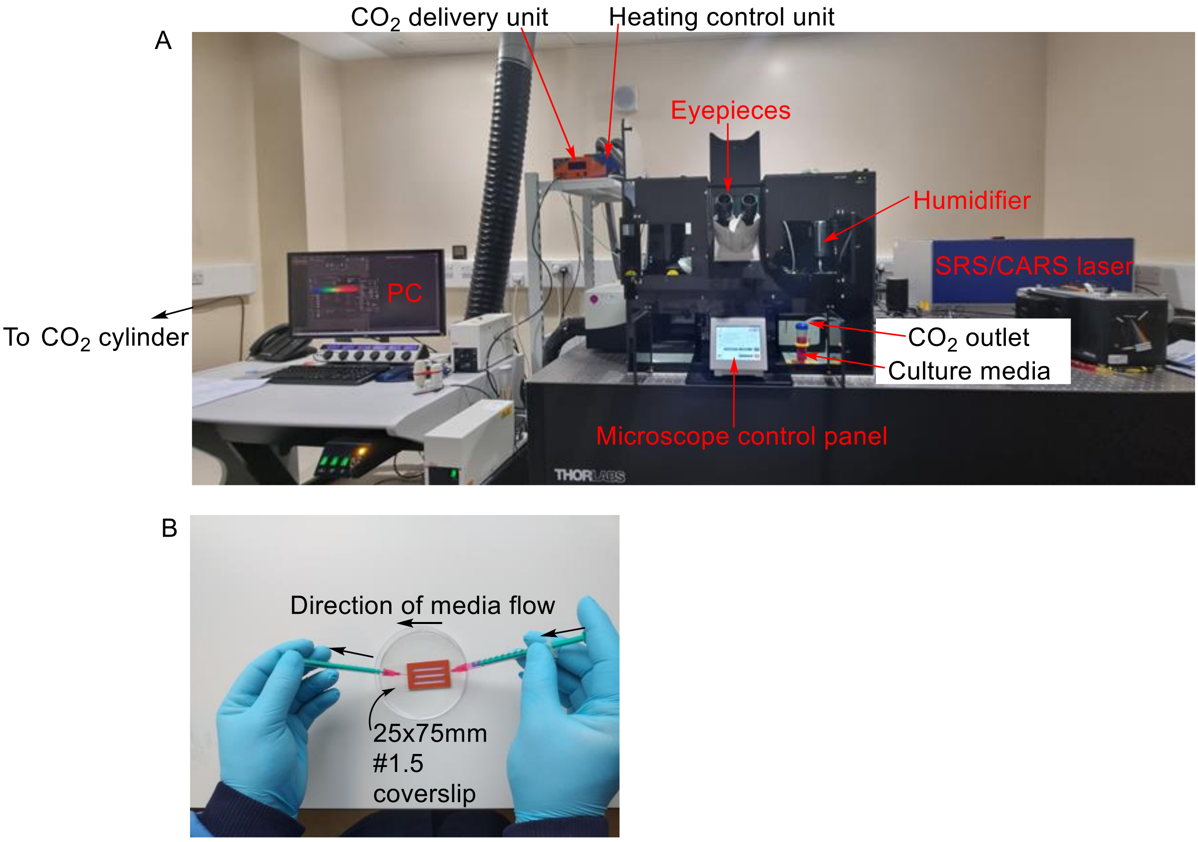

Figure S3 The perfusion chamber set-up and imaging system. **A** Multimodal SRS microscope at the University of Strathclyde used for live cell imaging studies. **B** Perfusion chambers used in this study. The chambers are adhesive and are attached to a coverslip. The channel depth of the chambers used in this study was 0.1 mm which contains around 80 µL media, or alternatively, where long-term imaging was performed, a channel depth of 0.6 mm (400 µL media) was used. Product details are available online: https://gracebio.com/products/imaging-microscopy/coverwell-perfusionchambers-imaging/; accessed 30th May 2022. Only one channel of the 3-channel systems was used for imaging.

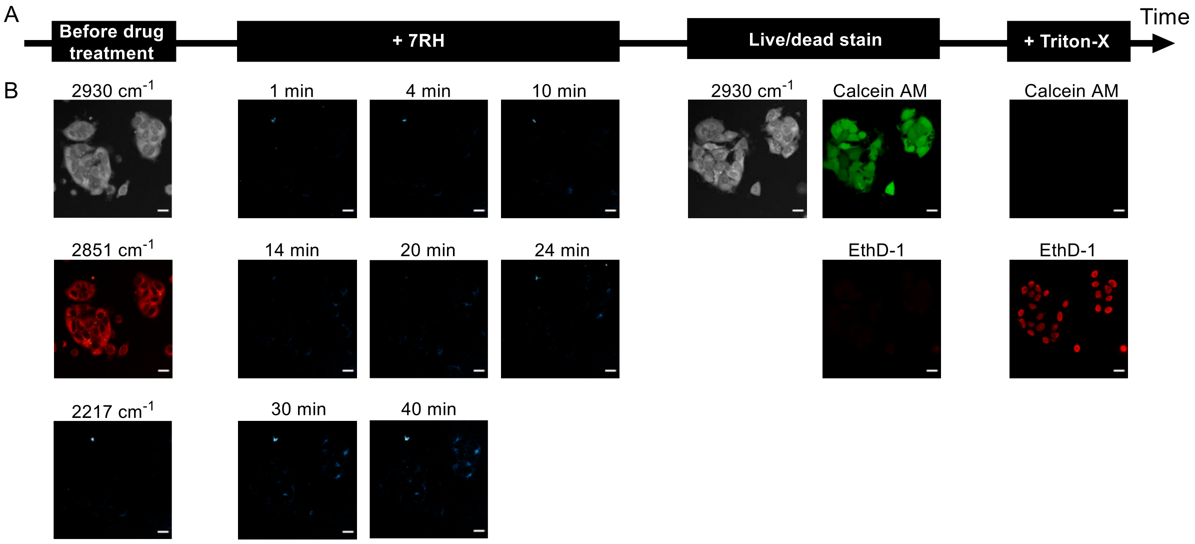

Figure S4 Perfusion experiment using multimodal imaging and 7RH. **A** Schematic workflow describing cell culture under media exchange conditions. **B** SRS images were acquired at 2930 cm^{-1} (CH_3 symmetric stretch), 2851 cm^{-1} (CH_2 symmetric stretch), 2117 cm^{-1} (cell-silent region) and 2217 cm^{-1} (C≡C, 7RH) prior to treatment with 7RH. The cells were then treated with 7RH in RPMI media (1 μM) and SRS images were acquired at 2217 cm^{-1} every 2 minutes for 40 minutes. Representative images are provided at the indicated timepoints. The cells were then washed with PBS prior to imaging at 2930 cm^{-1} and staining with a solution of Calcein AM (λ_{ex} = 488 nm; λ_{em} = 495-525 nm, live cells) and ethidium homodimer-1 (EthD-1, λ_{ex} = 514 nm; λ_{em} = 535-650 nm, dead cells) in PBS. The cells were then washed with PBS before permeabilization with Triton-X and subsequent staining with Calcein AM and EthD-1. Scale bars: 20 μm.

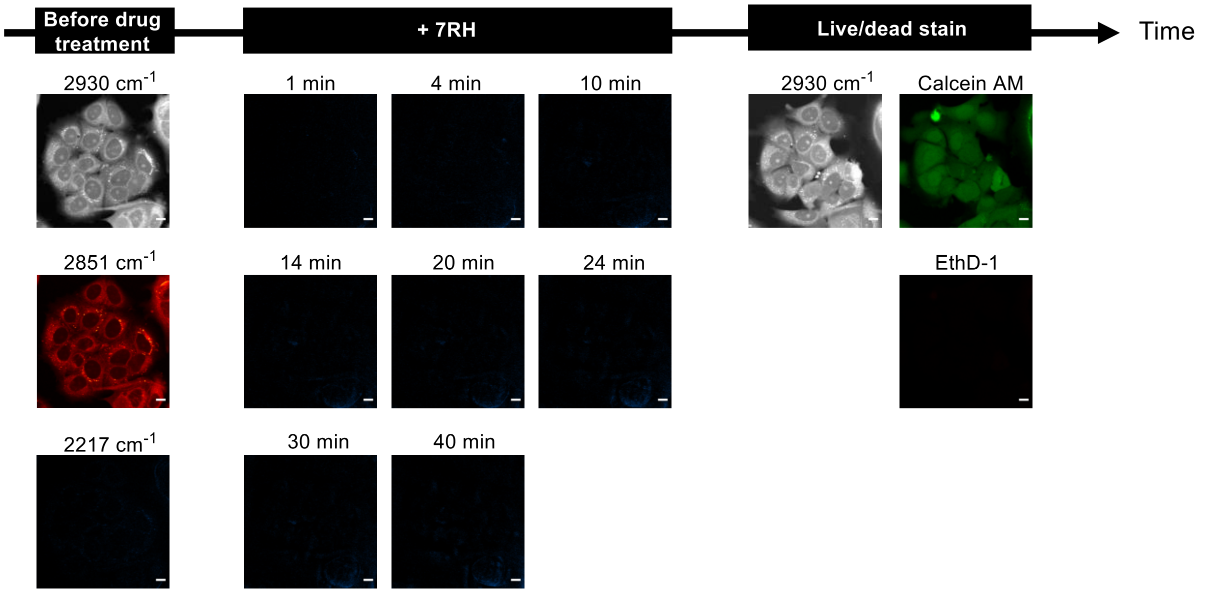

Figure S5 Control perfusion experiment using multimodal imaging. Schematic workflow describing cell culture under media exchange conditions. SRS images were acquired at 2930 cm^{-1} (CH_3 symmetric stretch), 2851 cm^{-1} (CH_2 symmetric stretch), 2117 cm^{-1} (cell-silent region) and 2217 cm^{-1} (C≡C, 7RH) prior to treatment with 7RH. The cells were then treated with RPMI + DMSO (0.1% v/v) and SRS images were acquired at 2217 cm^{-1} every 2 minutes for 40 minutes. Representative images are provided at the indicated timepoints. The cells were then washed with PBS prior to imaging at 2930 cm^{-1} and staining with a solution of Calcein AM (λ_{ex} = 488 nm; λ_{em} = 495-525 nm, live cells) and ethidium homodimer-1 (EthD-1, λ_{ex} = 514 nm; λ_{em} = 535-650 nm, dead cells) in PBS. Scale bars: 20 µm.

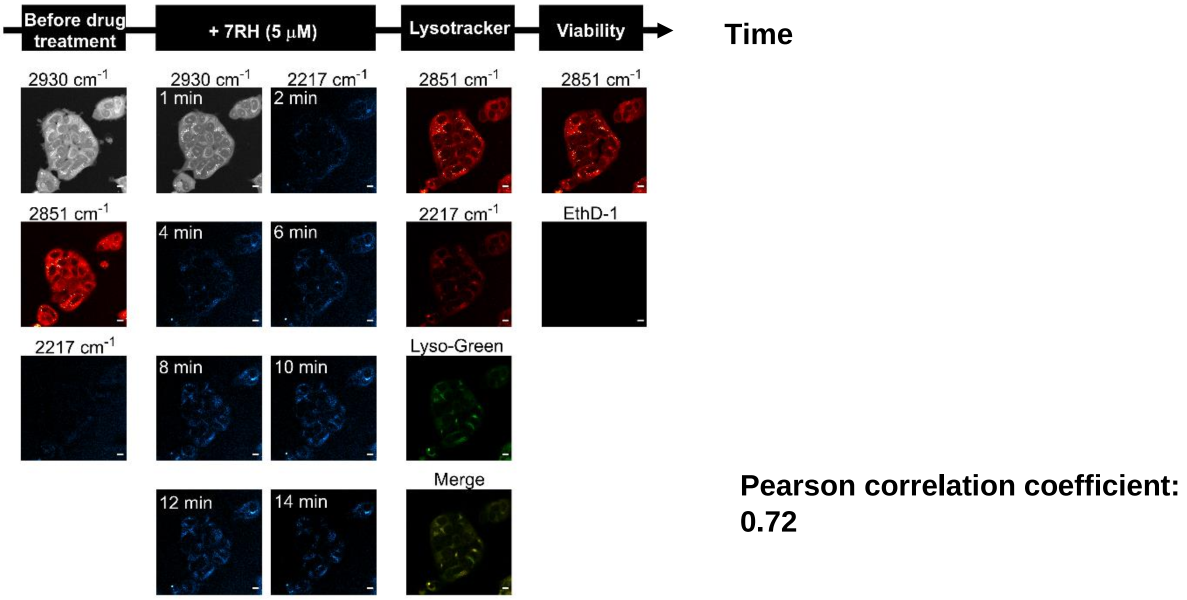

Figure S6 Perfusion experiment using multimodal imaging and 7RH. Schematic workflow describing cell culture under media exchange conditions. SRS images were acquired at 2930 cm^{-1} (CH_3 symmetric stretch), 2851 cm^{-1} (CH_2 symmetric stretch), and 2217 cm^{-1} (C≡C, 7RH) prior to treatment with 7RH (5 μM). The cells were then treated with 7RH in RPMI media (5 μM) and SRS images were acquired at 2217 cm^{-1} every 2 minutes for 14 minutes. Representative images are provided at the indicated timepoints. The cells were then washed with PBS prior to imaging at 2851 cm^{-1}, 2217 cm^{-1} (7RH, C≡C, red) and LysoTracker Green (λ_{ex} = 488 nm; λ_{em} = 495-525 nm, lysosomes). A merge image of the 2217 cm^{-1} (7RH, C≡C, red) and LysoTracker Green is also presented. Lastly, the cells were washed with PBS before staining with ethidium homodimer-1 (EthD-1, λ_{ex} = 514 nm; λ_{em} = 535-650 nm, dead cells) in PBS, indicating the cells are still viable. Scale bars: 20 μm.
